# Structure of human dipeptidyl peptidase 10 (DPPY): a modulator of neuronal Kv4 channels

**DOI:** 10.1038/srep08769

**Published:** 2015-03-05

**Authors:** Gustavo Arruda Bezerra, Elena Dobrovetsky, Alma Seitova, Sofiya Fedosyuk, Sirano Dhe-Paganon, Karl Gruber

**Affiliations:** 1 Institute of Molecular Biosciences, University of Graz, Humboldtstraße 50/3, A-8010 Graz, Austria; 2 Department of Physiology and Structural Genomics Consortium, University of Toronto, MaRS Centre, South Tower, 101 College St., Suite 700, Toronto, ON, M5G 1L7, Canada; 3 Max F. Perutz Laboratories, Medical University of Vienna, Dr. Bohr-Gasse 9/3, A-1030 Vienna, Austria

## Abstract

The voltage-gated potassium channel family (Kv) constitutes the most diverse class of ion channels in the nervous system. Dipeptidyl peptidase 10 (DPP10) is an inactive peptidase that modulates the electrophysiological properties, cell-surface expression and subcellular localization of voltage-gated potassium channels. As a consequence, DPP10 malfunctioning is associated with neurodegenerative conditions like Alzheimer and fronto-temporal dementia, making this protein an attractive drug target. In this work, we report the crystal structure of DPP10 and compare it to that of DPP6 and DPP4. DPP10 belongs to the S9B serine protease subfamily and contains two domains with two distinct folds: a β-propeller and a classical α/β-hydrolase fold. The catalytic serine, however, is replaced by a glycine, rendering the protein enzymatically inactive. Difference in the entrance channels to the active sites between DPP10 and DPP4 provide an additional rationale for the lack of activity. We also characterize the DPP10 dimer interface focusing on the alternative approach for designing drugs able to target protein-protein interactions.

The most diverse class of ion channels in the nervous and cardiovascular system is the voltage-gated potassium channel family (Kv) consisting of 12 subfamilies (Kv1–Kv12). A large number of regulatory proteins interact with the pore forming α-subunits of these channels^1^,^2^. Kv4 is a highly conserved subfamily of voltage-gated potassium channels, members of which are expressed in the soma and dendrites of central neurons^3^ and modulate A-type potassium currents (I_sa_). This family of ion channels regulates the propagation of action potential, firing frequency and synaptic integration/plasticity, and has therefore been implicated in neuronal and heart disorders^4^. Several spider toxins were shown to selectively inhibit Kv4 channel currents by modifying their gating kinetics^5^. The Ca^2+^ binding proteins (KChIPS) and the dipeptidylpeptidase-like proteins (DPPLs) DPP6 (also known as DPPX) and DPP10 (also known as DPPY) associate with the α-subunits of the Kv4 subfamily to form a ternary complex of approximately 750 kDa containing 12 protein molecules (4 copies of each protein per channel)^6^. In the current model, pore forming subunits of Kv4 subfamily are embedded in the membrane, with KChIPs assembling at the cytoplasmic side and DPPLs at the extracellular side (Fig. S1)^7^. These associated subunits play an important regulatory role by modulating the electrophysiological properties, cell-surface expression and subcellular localization of the channels.

In 2003, DPP6 was co-purified with A-type potassium channel complexes from rat brain membranes. The protein was shown to regulate the channel subunit's trafficking, membrane targeting and function in somatodendritic compartments of neurons, and also to reconstitute the characteristics of native channels expressed in heterologous systems^8^. In the same year, DPP10 was cloned from a human hypothalamus cDNA library after its sequence was revealed by a Blast search^9^ using the full length sequences of human fibroblast activation protein (FAP) and DPP4 (also known as CD26)^10^. Structurally related to DPP4, DPP6 and DPP10 are members of the prolyl oligopeptidase family (both sharing approximately 30% sequence identity with DPP4) and belong to the S9B serine protease subfamily. DPP4 is the archetype of this family and its best characterized member, as it is implicated in a myriad of physiological processes, including development of cancer^11^ and glucose metabolism and it is targeted by the latest generation drugs for the treatment of type 2 diabetes^12^.

DPP6 and DPP10 are homologous glycosylated, single-pass type II transmembrane proteins that lack the crucial serine residue of the catalytic triad; instead, DPP6 contains an aspartic acid (Asp712) and DPP10 a glycine (Gly651). Attempts to recover enzymatic activity by means of site directed mutagenesis, where the aspartic acid in DPP6 and the glycine in DPP10 were mutated to serine, were not successful^13^,^14^. Clearly, the molecular roles of these proteins are unrelated to serine protease catalytic activity.

Although DPP6 is broadly expressed^15^, DPP10's restricted expression, including the brain, adrenal gland and pancreas, may serve as a marker in certain malignant states such as colorectal cancer and could have a prognostic significance^16^. The presence of DPP10 in endocrine cells suggests that the protein may also have an additional function related to the regulation of hormone secretion^1^. DPP10 and DPP6 both accelerate the activation and inactivation of Kv4.3 channel gating but with distinct biophysical properties^1^. For instance, differences in the recovery kinetics from an inactivated state have been observed in Kv4.2 channels co-expressed with either of the two proteins in *Xenopus* oocytes and CHO cells^17^. This disparity in modulation is very likely to be central to the correct propagation of neuronal somatodendritic A-type potassium currents. While it is well established that the residues at the N-terminus and transmembrane region play a fundamental role in the complex formation and modulation of the channels, the exact function of the extracellular domain remains largely unknown^1^,^18^. Recently it was shown that glycosylation at the extracellular domain of DPP10 is required for proper cell surface sorting and formation of the ternary channel complex between DPP10 and Kv4.3/KChIP2a^19^.

Since DPP6 and DPP10 are components of the neuronal Kv4 channels, these proteins are potential drug targets for diseases such as Parkinson's, schizophrenia and temporal lobe epilepsy^20^,^21^. Indeed, aggregation of DPP10 was associated with neurodegenerative conditions such as Alzheimers, diffuse Lewy body disease and fronto-temporal dementia^22^. Polymorphisms in the genes encoding DPP10 and DPP6 are associated with asthma and amyotrophic lateral sclerosis, respectively^23^,^24^. Additionally, recent studies show that deletions and duplications in the DPP10 gene are enriched in people with autism indicating the importance of this protein for proper cognitive function^25^. In order to better understand the role of these proteins in these diseases and to evaluate these targets for therapy, a better understanding of their molecular function is needed. Because knowledge of 3D structures can be particularly insightful in determining the molecular basis of protein function, we determined the crystal structure of the extracellular domain of DPP10.

## Results and Discussion

### Overall structure

In this study, a DPP10 variant corresponding to GenBank entry AAH30832.1, obtained from a Mammalian Gene Collection cDNA template^26^, was truncated lacking the N-terminal cytoplasmic (residues 1–34) and transmembrane domains (residues 35–55). The extracellular domain (residues 58–796) was expressed as a secreted protein from insect cells, purified and crystallized in space group *P*2_1_2_1_2_1_ with two molecules in the asymmetric unit. The structure was solved at 3.4 Å resolution by molecular replacement using the structure of human DPP6 (PDB code: 1XFD)^27^ as template, which shares close to 50% sequence identity with DPP10. The model was then refined to a working R-factor of 20.7% and an R_free_ 24.1%. Interpretable electron density was present for residues 65 to 783. Detailed statistics of the structure are listed in Table 1.

**Figure 1:**
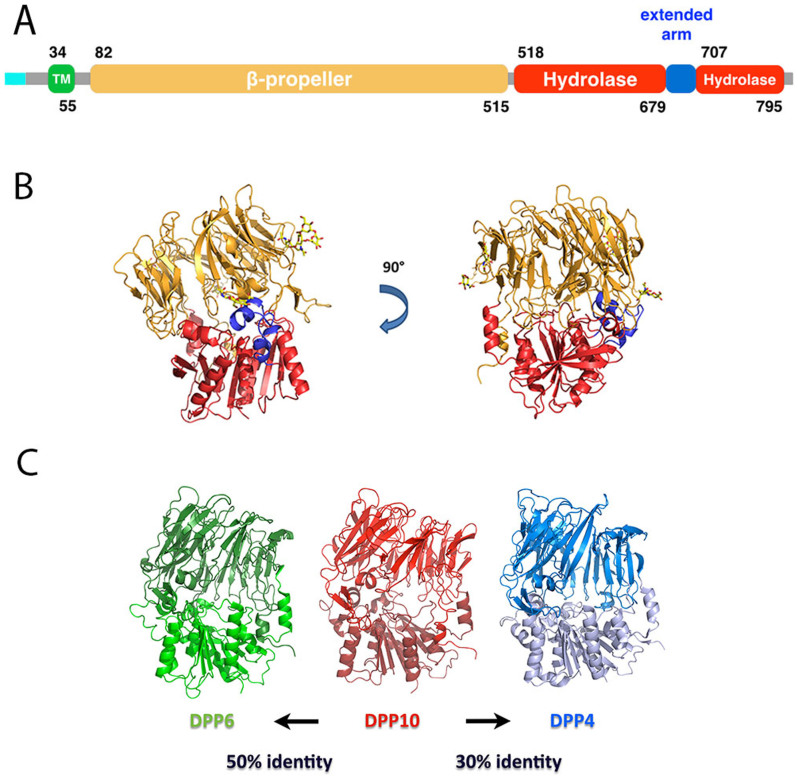
Overall structure of DPP10. (A) Scheme displaying the domain organization of DPP10. The domains are represented by boxes: the cytoplasmic domain is shown in cyan, the transmembrane domain in green, the β-propeller in yellow, the α/β-hydrolase domain in red and the extended arm in blue. (B) Cartoon representation of the structure of DPP10 using the same color code as in panel A. Sugar residues are shown as yellow sticks. (C) Comparison of the structures of DPP10 (light red), DPP6 (blue) and DPP4 (yellow) each in a cartoon representation.

The overall structure of DPP10 is very similar to those of DPP6 and DPP4 (Fig. 1), with Cα-root-mean-square-deviations (r.m.s.d.) of 1.1 Å (for 646 superimposed atoms) and 1.5 Å (for 578 atoms), respectively. Each subunit consists of two domains: an open (no “Velcro” architecture present) β-propeller (residues 82–517, shown in yellow in Fig. 1) consisting of eight blades each built up by four antiparallel β-strands and a classical α/β-hydrolase fold (residues 517–679 and 707–796; shown in red). Additionally, a small-extended arm formed by three helices is inserted between two β-strands of the hydrolase domain (residues 679–707; shown in blue).

It has been shown previously that DPP10 possesses six N-glycosylation sites (residues 90, 111, 119, 257, 342 and 748), all of them except Asn-748 located in the β-propeller^19^. In our structure, all these asparagine residues with the exception of Asn-90 were found to be glycosylated and well-defined electron densities were observed for the first sugar residues. Because DPP10 was expressed in SF9 insect cells, we modeled the carbohydrates based on the Asn-β1-GlcNac-β1,4-GlcNac-β1,4-Man-α1,3-Man motif, as it is often the final glycan structure found in insects^28^. Similarly to DPP6, DPP10 has three disulfide bonds in the β-propeller (Cys349-Cys356, Cys465-Cys468, Cys475-Cys493) and one in the α/β-hydrolase domain (Cys670-Cys780).

### Active site and access channel

**Figure 2:**
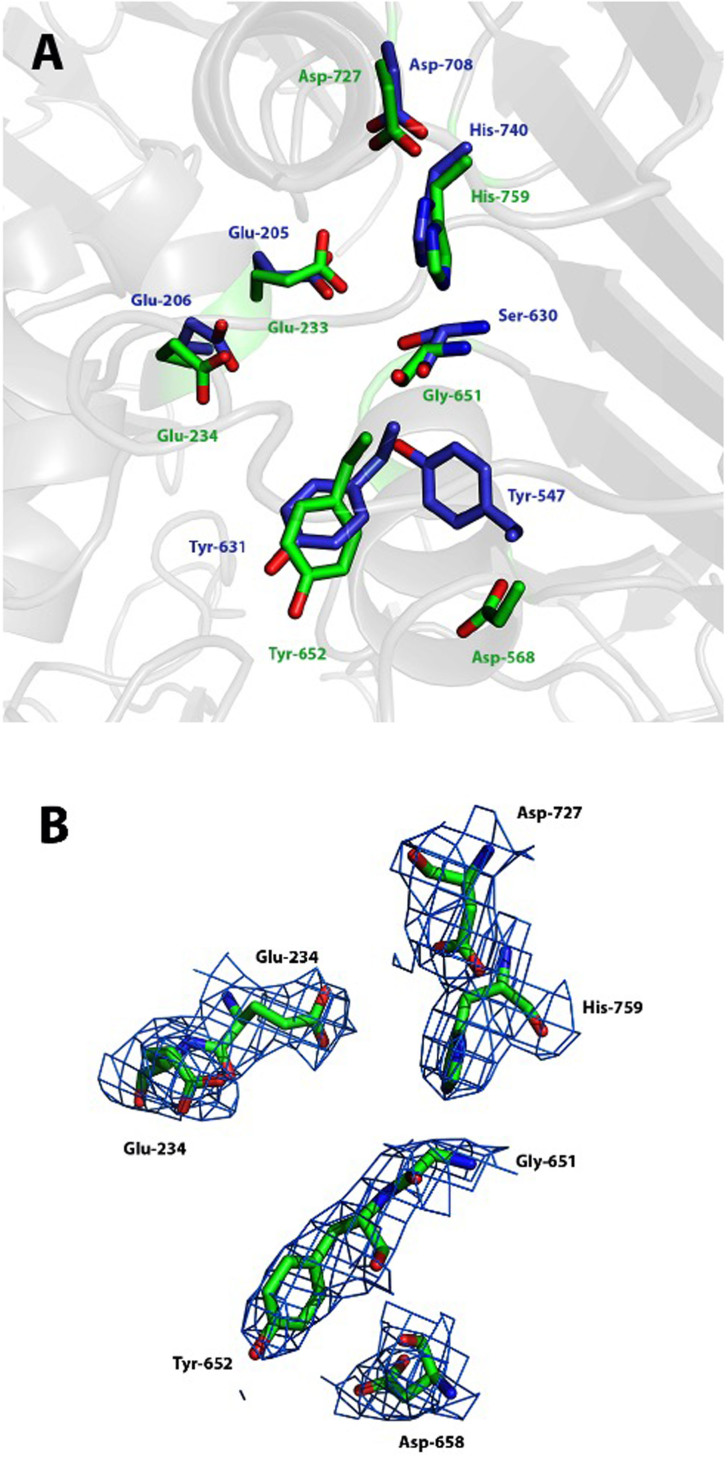
“Catalytic triad” of DPP10. (A) Superposition of DPP10 (shown in green) “catalytic triad” (Gly-651, His-759 and Asp-727), N-anchor glutamates (Glu-233 and Glu-234) and oxyanion holes (Asp-568 and Tyr 652) to that of DPP4 (shown in blue, PDB-code: 3EIO). (B) DPP10 “active site” 2Fo-Fc map contoured at 1σ is shown in blue. Side chains of the residues are shown as sticks.

Within the α/β-hydrolase fold, DPP4 contains the catalytic triad Ser-His-Asp, the conserved GWSYGG motif (which includes the catalytic serine), and a pair of highly conserved glutamate residues (that act as the N-anchor for the incoming peptide and are essential for enzyme catalysis)^29^. DPP10 contains the glutamate residues (Glu-233 and Glu-234), but the serine is replaced by a glycine (Fig. 2) and the tryptophan residue preceding the serine is replaced by a lysine (changing the sequence to GKGYGG). Thus, the residues analogous to the catalytic triad in DPP4 would be Gly-651, His-759 and Asp-727 in DPP10. Attempts to recover peptidase activity by engineering single (Gly to Ser), double (LysGly to TrpSer), or triple (Asp-568 by tyrosine) replacement variants were not successful^14^. The latter aspartate residue occupies the equivalent position of Tyr-547 in DPP4, which was shown to be essential for DPP4 activity^30^. Similarly, replacement of the active site aspartate in DPP6 by serine did not recover peptidase activity^13^,^14^.

The basis of DPP6/10 catalytic inactivity is not fully understood. Despite the absence of the catalytic serine residue in DPP10, the triad histidine and the aspartate form a hydrogen bond, and because the conformation of the nucleophile elbow (GXSXG) is unaltered in DPP10 compared to DPP4 (Fig. 2), it is conceivable that the Gly to Ser amino acid exchange produces a structurally intact Ser-His-Asp catalytic triad. The fact that the engineered variants did not show any peptidase activity suggests that other necessary structural features are missing in DPP10 (and DPP6).

**Figure 3:**
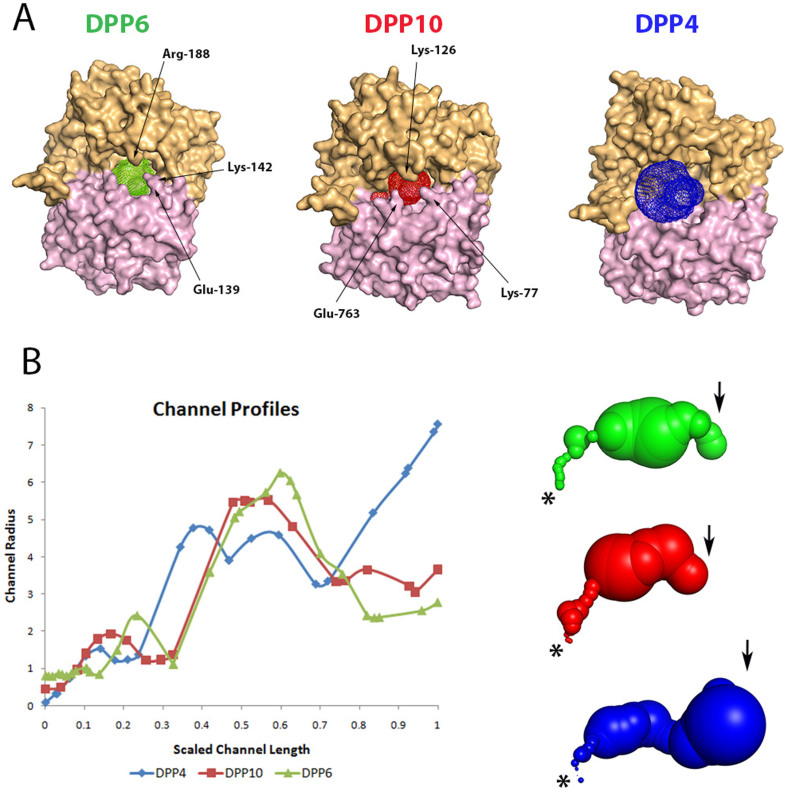
Comparison of the “active site” access channel in DPP4, DPP10 and DPP6. (A) Surface representation of DPP6, DPP10 and DPP4 with the channels shown in green, red and blue, respectively. The β-propeller domain is colored in orange and the α/β-hydrolase domain in pink. (B) Channel profiles indicating the radius in Å *vs.* the scaled length, starting from the “catalytic triad” position (indicated by a star) towards the protein surface (indicated by an arrow). Surface representation of the channels using the same coloring scheme as in panel A.

We hypothesized that for enzymatically inactive proteins there should not be evolutionary pressure against the anatomy of the access channel to the “active site”, and therefore we compared the geometry of the corresponding channels of DPP10 and DPP6 to that of DPP4 using the program Caver^31^. For DPP4 it is well established that substrates enter the active site through a big channel between the hydrolase and β-propeller domain, and the products very likely leave through a channel in the center of the β-propeller domain (Fig. 3)^29^. In DPP10, the entrance is narrowed by the presence of Lys-77, Lys-126 and Glu-763 (Fig. 3A, channel colored in red). The residues Lys-76 and Glu-763 originate from the hydrolase domain, while Lys-126 originates from the β-propeller domain. In DPP6, the residues Glu-139, Lys-142 and Arg-188 (Fig. 3A, channel colored in green) are responsible for the blockage of the channel. The way these residues are arranged considerably narrows the channel entrance radius to 3.5 Å and 4 Å in DPP10 and DPP6, respectively. In DPP4 this radius is two-fold larger at 7.5 Å (Fig. 3B). To validate our hypothesis, we performed *in silico* mutations in DPP10 (K77A/K126A/E763A) and DPP6 (E139A/K142A/R188A). In both cases, the triple mutants showed opening of the entrance channel to the “active site” (Fig. S3). Therefore, we hypothesize that narrowing of the substrate access route to the catalytic triad (entry of substrates) prevents accidental imprisonment of random molecules in the binding pocket of the protein. Moreover, our analysis provides additional insights to the fact that catalytic-recovery-type mutations failed probably because the access channel for the substrates was blocked.

### Dimerization Interface

**Figure 4:**
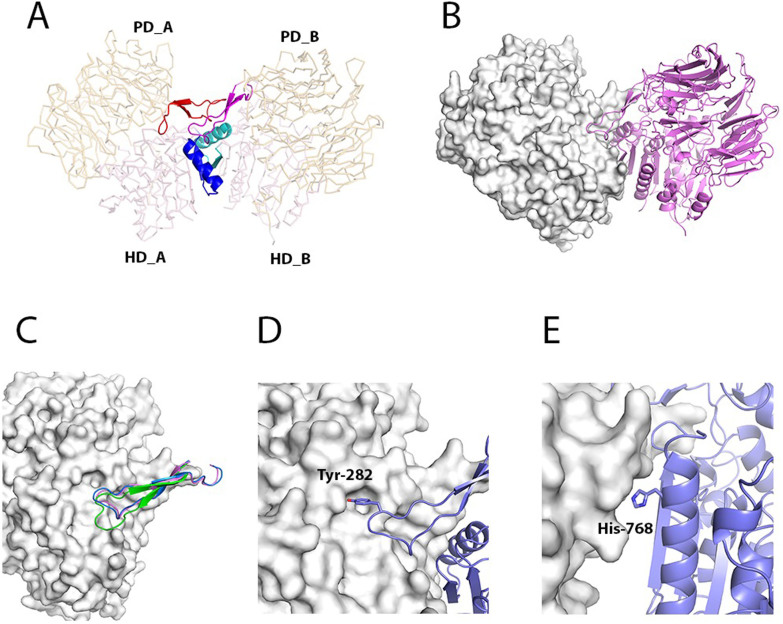
Dimerization interface. (A) Cartoon representation of residues participating in the dimer interface. The β-propeller domains are indicated as PD_A and PD_B, and the α/β-hydrolase domains as HD_A and HD_B in subunits A and B, respectively. The β-propeller loops are colored in red and magenta, and the C-terminal helices in blue and cyan for the subunits A and B, respectively. (B) Heterodimer model of DPP10 (surface representation) and DPP6 (cartoon representation). This model was generated by superimposing DPP6 chain A onto DPP10 chain B. (C) Cartoon representation of β-propeller loop of DPP10 (blue), DPP6 (magenta) and DPPIV (green) superimposed to the opposite subunit of DPP10 (surface representation). (D) Interaction of Tyr-282 with the opposite subunit A in DPP10 and (E) His-768.

According to a PISA analysis^32^ the two DPP10 molecules in the asymmetric unit of the crystal form a symmetric dimer with a total buried surface area of 7300 Å^2^. The main dimerization interface, which has a two-fold symmetry, between the two chains (1580 Å^2^ per chain) is formed primarily by residues Pro-262 to Gly-277 and Tyr-282 to Gln-287 of the β-propeller domain, as well as by residues Phe-732 to Ala-745 and Thr-750 to Pro-755 located in the hydrolase domain (Fig. 4A). These residues interact with their equivalents in the opposite subunit. Interestingly, theses stretches of interfacing residues are highly conserved among DPP4-like proteins and have been named “β-propeller loop” (for the residues in the β-propeller domain) and “C-terminal loop” (for the residues in the hydrolase domain)^33^,^34^.

DPP4 and DPP6 have been shown to form dimers in solution^19^,^27^. The basis of homodimerization has been attributed to a 2190 Å^2^ patch on DPP4 that encompasses the β-propeller and C-terminal loops (PDB-code: 3EIO)^35^. Analogous interactions are seen in DPP6 (PDB-code: 1XFD) and now also in our DPP10 structure, which buries 1580 Å^2^. DPP4 forms 10 inter-chain salt bridges and 30 hydrogen bonds. Because the DPP6 interface features 22 hydrogen bonds and the DPP10 interface 13 hydrogen bonds, with none of them containing any salt bridges, these interactions may be weaker than that of DPP4.

The comparison of the DPP10 dimerization interface with DPP6 supports the finding that the two proteins are able to form heterodimers^1^. A superposition of the β-propeller loop (Cα-r.m.s.d. of 0.59 Å over 23 atoms) indicates the compatibility of this region for hetero oligomerization (Fig. 4B). In general, residues at the dimer interface appear to be more conserved between DPP10 and DPP6 (62% identity in the interface *vs.* 50% overall identity)^27^. There are differences in the amino acid sequences of the β-propeller loop, but the most dissimilar residues regarding side chain bulkiness or polarity are more exposed to the solvent and contribute less to the interface: Val-265, Arg-268, Lys-276, Gln-287 in DPP10 are equivalent to Glu-327, Thr-330, Thr-338 and Ser-349 in DPP6, respectively. On the other hand, superposition of DPP4 with either DPP10 or DPP6 indicates that heterodimers are less likely to form due to steric clashes caused by two additional residues in the β-propeller loop of DPP4 (Fig. 4C).

Residues in the second major dimerization region – the C-terminal loop – are even more conserved between DPP10 and DPP6 than those in the β-propeller loop. A superposition of this region results in a Cα-r.m.s.d. of 0.48 Å over 14 atoms. In comparison to DPP4, however, this area is responsible for the most discrepancy regarding dimer interactions. In DPP4, 23 residues directly participate in the dimer formation, compared to 14 residues in DPP10 and 11 residues in DPP6.

Could small molecules that disrupt the dimer interface^36^ be useful tool compounds to test the importance of this interaction in disease models and possibly lay the groundwork for future therapies? Tyr-282 and His-768, both highly conserved among prolyl-oligoppetidases, act as anchor residues occupying a pocket in the opposite subunit (Fig. 4D and 4E). In DPP4, it was shown that exchanging the histidine by a glutamate completely abolishes dimerization^33^. Also, the equivalent tyrosine to alanine mutant prevents intermolecular interactions in DPP4, resulting in the monomeric form of the enzyme^34^. Small molecules targeting this pocket could potentially prevent dimer formation. Because drugs that oppose KV4 inactivation are assumed to be useful for treating pathological hyperexcitability related to disorders such as epilepsy and asthma^24^,^37^, and DPP10 and DPP6 confer fast inactivation of *I_sa_*, inhibition of these molecules by dimer disruption could lead to an alternative treatment of those conditions.

Subtle differences within the protein-protein interface of these molecules may represent potential hotspots for the development of small molecules that could selectively disrupt/stabilize specific oligomers, which could provide a new way to regulate DPPs activities.

### Glycosylation

**Figure 5:**
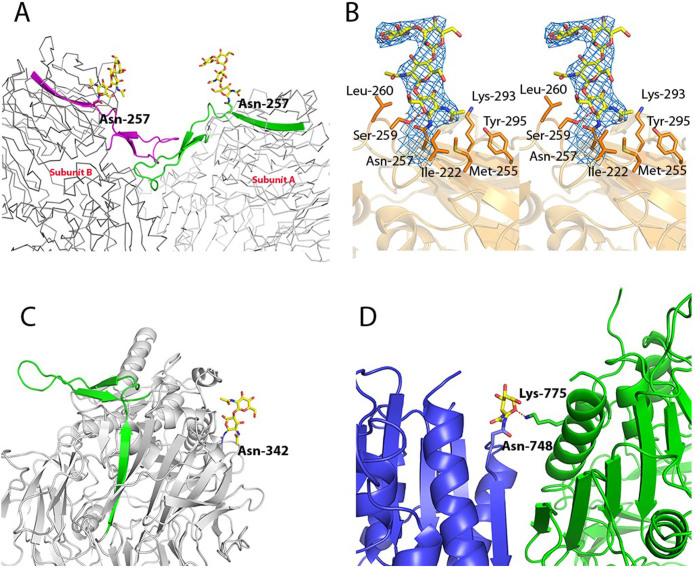
DPP10 glycosylation. (A) The glycosylated Asn-257 is placed adjacent to the β-propeller loop, and might stabilize it. Both subunits are represented as ribbons, the β-propeller loops as cartoons and Asn-257 is shown as sticks. Subunit A is shown in light grey and green, while subunit B is shown in dark grey and magenta. (B) Interactions between the carbohydrate and the residues in the subunit A. Ser-229 makes a hydrogen bond with O5 (possibly O6 in subunit B) while Leu-222, Met-255, Leu-260, Tyr-295 and Lys-293 contribute with hydrophobic interactions. Glycan 2Fo-Fc map contoured at 1σ is shown in blue. (C) The glycosylation at Asn-342 might indirectly stabilize the β-propeller loop, since it is positioned relatively close to this region. The protein is shown as light grey cartoon and the β-propeller loop is shown as green cartoon. (D) N-acetylglucosamine bound to Asn-748 in the subunit B (shown as dark grey cartoon) possibly interacts through a hydrogen bond with Lys-775 of subunit A (shown as light grey cartoon). The carbohydrates are shown as yellow sticks and the hydrogen bond as red dashes.

Recent studies demonstrated that inhibition of glycosylation by tunicamycin reduced the protein cell surface expression and affected the modulation of Kv4.3-mediated transient outward potassium current (I_to_) inactivation and recovery from inactivation^38^. In site-directed mutagenesis experiments (Asn replaced by Gln) at the predicted glycosylation sites each variant showed reduced cell surface expression levels compared to wild type DPP10^19^. Our DPP10 crystal structure offers some insights into these results. For instance, Asn-257 which is close to the dimer interface, located adjacent to the β-propeller loop (Fig. 5A), when exchanged Asn257Gln completely abolished cell surface trafficking and dimer formation. Glycosylation at this position might play a role in dimerization by stabilizing the β-propeller loop. Ser-259 makes a hydrogen bond with O5 (in subunit A) and possibly O6 (in subunit B) of the first carbohydrate moiety. Ser-259 and the first carbohydrate moiety of Asn-257 glycan present B-factor of 50.5 Å^2^ and 74.2 Å^2^, respectively; the structure average B-factor is 76.3 Å^2^. The residues Ile-222, Met-255, Leu-260, Lys-293, and Tyr-295 additionally contribute with hydrophobic interactions (Fig. 5B). Glycosylation at Asn-342 was also shown to be important for dimer formation^19^. This residue is also located in the vicinity of the dimer interface area and might stabilize necessary elements for the maintenance of the local conformation (Fig. 5C). Interestingly, the N-acetylglucosamine moiety (B-factor 189.9 Å^2^) attached to Asn-748 (B-factor 124.8 Å^2^) possibly interacts with Lys-775 (B-factor 95.6 Å^2^) from the other subunit (Fig. 5D) and likely also plays a role in dimer stabilization. DPP6 has a slightly different glycosylation pattern compared to that of DPP10, which could be related to the differences observed in the electrophysiological properties of Kv4 channels formed by each of the proteins^17^. For instance, Asn-90 and Asn-119 in DPP10 have no equivalent in DPP6, while Asn-471, Asn-535 and Asn-566 in DPP6 have no equivalent in DPP10. In this way, DPP6 has one additional glycosylation site that could represent the basis of its specific molecular effect on the ion channels.

## Conclusion

In this paper, we present the crystal structure of human DPP10, a naturally inactive member of the prolyl oligopeptidase family that belongs to the S9B serine protease subfamily. We provide the structural basis for its inherent inactivity, oligomeric association and glycosylation state, which are features required for the proper functioning of the protein. We envisage that our DPP10 crystal structure will support studies to elucidate the role of these molecules in the modulation of Kv4 channels, how it differs from that of DPP6 and its relationship with neuronal disorders. Moreover, our structure offers a starting point for the design of specific molecules targeting protein-protein interactions in the dimer interface.

## Methods

### Protein cloning, expression and purification

The cloning, expression and purification of DPP10 was previously described in detail^39^. The gene for human DPP10 was obtained from a Mammalian Gene Collection cDNA template (AT23-C8) and ligated into the pFHMSPLICN plasmid (a modified pFastBac™HT A vector (Invitrogen)). The resulting plasmid SDC134F03 was transformed into DH10Bac™ competent *Escherichia coli* cells (Invitrogen) and a recombinant viral DNA bacmid was purified followed by a recombinant baculovirus generation in Sf9 insect cells. The Sf9 cells grown in HyQ SFX insect serum-free medium (TermoScientific) were infected with the respective amount of recombinant baculovirus and incubated for 3–4 days at 27°C using a platform shaker set at 100 rev min^−1^.

For protein purification, the culture medium was centrifuged at 14000 g for 15 min and the pH of the supernatant was adjusted to 7.5 at 25°C by titrating with 50 mM Tris-HCl, pH 8.0 and 1.5 M NaCl. The culture medium was loaded onto Ni-NTA Superflow beads (Qiagen) and washed with 10 CV of washing buffer containing 50 mM Tris-HCl, pH 8.0, 500 mM NaCl and 5 mM imidazole. The protein was eluted with the elution buffer composed of 50 mM Tris-HCl, pH 8.0, 500 mM NaCl, 300 mM imidazole and 5%(v/v) glycerol. Further purification was performed by size-exclusion chromatography (SEC) on a HiLoad 16/60 Superdex 75 (GE Healthcare) column equilibrated with 50 mM Tris-HCl, pH 8.0 and 100 mM NaCl. The N-terminal His-tag was removed by proteolysis with the TEV protease at a TEV:DPP10 ratio of 1:50 and subsequent passage of the sample through a 1 ml HisTrap FF crude column (GE Healthcare) equilibrated with the buffer used for SEC.

### Crystallization

Crystallization trials were carried out at the Structural Genomics Consortium (SGC) high-throughput platform in Toronto using the sitting-drop vapour-diffusion method in a 96-well Intelli-Plate (ArtRobbins Instruments) at 293 K by mixing equal volumes (0.5 μL) of 2 mg·ml^−1^ protein and reservoir solution using a Mosquito robot (TTP LabTech). Crystallization trials were initially performed using the in-house screens RedWing and SGC-I. Each screen consists of 96 conditions that were elaborated from commercial and published screens priorizing conditions with the highest known success rates. The detailed formulations of these screens can be found on the SGC website (http://www.sgc.utoronto.ca/SGC-WebPages/toronto-technology-crystallization.php). Crystals formed within 2–4 weeks using 100 μL of reservoir solution consisting of 20%(w/v) PEG 1500 (Sigma–Aldrich), 0.2 M MgCl_2_, 0.1 M sodium cacodylate pH 5.5. Most DPP10 crystals possessed rectangular shapes, with average dimensions of about 0.1 × 0.05 × 0.005 mm (as estimated based on comparison with the 0.1 mm cryoloop used for crystal mounting). Crystals were flash-cooled in liquid nitrogen after being cryoprotected by dipping it in a solution of 25%(w/v) PEG 1500, 0.2 M MgCl2, 0.1 M sodium cacodylate pH 5.5, 10%(v/v) glycerol. Optimization of the crystallization conditions did not result in improved diffraction patterns.

### Crystal structure determination

Diffraction data were collected on beamline 19-ID at the Advanced Photon Source (APS), Chicago, USA and were processed using the programs MOSFLM^40^ and SCALA^41^. The phase problem was solved by molecular replacement using BALBES^42^ from the CCP4 package (CCP4, 1994)^43^. The BALBES pipeline produced a clear solution with two molecules in the asymmetric unit employing the structure of human DPP6^27^ (PDB code 1XFD) as search template. The initial R and R_free_ values were 0.454 and 0.444, respectively. The model was further refined by interactive adjustments using COOT^44^ and restrained refinement cycles in PHENIX^45^, employing non-crystallographic 2-fold symmetry, to R_cryst_ and R_free_ values of 0.207 and 0.241, respectively. Clear density was observed for residues 65–783. The final model was validated using MOLPROBITY^46^ which showed that 98.5% of amino acid residues are in the allowed region of the Ramachandran plot and displayed 12 outliers in chain A and 10 outliers in chain B. The coordinates and structure factors have been deposited with the PDB under accession code 4WJL. Details of data collection, processing and refinement are summarized in Table 1. The software CAVER was used for computing the protein channels^31^. The program PyMOL (http://www.pymol.org) was used for analysis of the 3D structures and figures preparation.

## Author Contributions

Conceived and designed the study: G.A.B., E.D., S.D.-P. and K.G. Performed the experiments: G.A.B., E.G. and A.S. Analyzed the data: G.A.B., S.F. and K.G. Wrote the manuscript: G.A.B., S.F. and K.G. All authors reviewed the manuscript.

## Additional information

**Accession codes**: The coordinates and structure factors have been deposited with the PDB under accession code 4WJL.

## Supplementary Material

Supplementary Information Supplementary information

## Figures and Tables

**Table 1:** Data collection and refinement statistics

	hDPP10
**Data collection**	
Beamline	APS 19-ID
Wavelength (Å)	0.97943
Unit cell	*A* = 80.9 Å *b* = 143.7 Å *c* = 176.2 Å
Space group	*P*2_1_2_1_2_1_
Resolution range (Å)*	66.5–3.4 (3.58–3.40)
Completeness (%)	99.9 (99.9)
Multiplicity	5.1 (5.0)
R_sym_	0.226 (0.775)
R_pim_	0.11 (0.378)
<I/σ_I_>	6.8 (2.3)
Unique reflections	29019
**Refinement**	
R/R_free_	0.207/0.241
r.m.s.-deviations	
bond length (Å)	0.008
bond angle (°)	1.36
Estimate coordinate error (Å)	0.37
Number of atoms	
protein	11600
carbohydrate	229
B-factors (Å^2^)	
protein	75.7
carbohydrate	107.5

*Values for the highest resolution shell are given in parentheses.

## References

[b1] Ren X., Hayashi Y., Yoshimura N. & Takimoto K. Transmembrane interaction mediates complex formation between peptidase homologues and Kv4 channels. Mol Cell Neurosci 29, 320–32 (2005). 15911355 10.1016/j.mcn.2005.02.003

[b2] Herson P. S. & Adelman J. P. It takes two to tango, but three to ISA. Neuron 37, 370–2 (2003). 12575944 10.1016/s0896-6273(03)00060-6

[b3] Covarrubias M. *et al.* The neuronal Kv4 channel complex. Neurochem Res 33, 1558–67 (2008). 18357523 10.1007/s11064-008-9650-8 PMC5833991

[b4] Amberg G. C., Koh S. D., Imaizumi Y., Ohya S. & Sanders K. M. A-type potassium currents in smooth muscle. Am J Physiol Cell Physiol 284, C583–95 (2003). 12556357 10.1152/ajpcell.00301.2002

[b5] Lundby A. *et al.* Effect of the I(to) activator NS5806 on cloned K(V)4 channels depends on the accessory protein KChIP2. Br J Pharmacol 160, 2028–44. 20649599 10.1111/j.1476-5381.2010.00859.x PMC2958647

[b6] Maffie J. & Rudy B. Weighing the evidence for a ternary protein complex mediating A-type K+ currents in neurons. J Physiol 586, 5609–23 (2008). 18845608 10.1113/jphysiol.2008.161620 PMC2655395

[b7] Soh H. & Goldstein S. A. I SA channel complexes include four subunits each of DPP6 and Kv4.2. J Biol Chem 283, 15072–7 (2008). 18364354 10.1074/jbc.M706964200 PMC2397469

[b8] Nadal M. S. *et al.* The CD26-related dipeptidyl aminopeptidase-like protein DPPX is a critical component of neuronal A-type K+ channels. Neuron 37, 449–61 (2003). 12575952 10.1016/s0896-6273(02)01185-6

[b9] Altschul S. F. *et al.* Gapped BLAST and PSI-BLAST: a new generation of protein database search programs. Nucleic Acids Res 25, 3389–402 (1997). 9254694 10.1093/nar/25.17.3389 PMC146917

[b10] Qi S. Y., Riviere P. J., Trojnar J., Junien J. L. & Akinsanya K. O. Cloning and characterization of dipeptidyl peptidase 10, a new member of an emerging subgroup of serine proteases. Biochem J 373, 179–89 (2003). 12662155 10.1042/BJ20021914 PMC1223468

[b11] Havre P. A. *et al.* The role of CD26/dipeptidyl peptidase IV in cancer. Front Biosci 13, 1634–45 (2008). 17981655 10.2741/2787

[b12] Feng J. *et al.* Discovery of alogliptin: a potent, selective, bioavailable, and efficacious inhibitor of dipeptidyl peptidase IV. J Med Chem 50, 2297–300 (2007). 17441705 10.1021/jm070104l

[b13] Kin Y., Misumi Y. & Ikehara Y. Biosynthesis and characterization of the brain-specific membrane protein DPPX, a dipeptidyl peptidase IV-related protein. J Biochem 129, 289–95 (2001). 11173531 10.1093/oxfordjournals.jbchem.a002856

[b14] Chen T. *et al.* Molecular characterization of a novel dipeptidyl peptidase like 2-short form (DPL2-s) that is highly expressed in the brain and lacks dipeptidyl peptidase activity. Biochim Biophys Acta 1764, 33–43 (2006). 16290253 10.1016/j.bbapap.2005.09.013

[b15] McNicholas K., Chen T. & Abbott C. A. Dipeptidyl peptidase (DP) 6 and DP10: novel brain proteins implicated in human health and disease. Clin Chem Lab Med 47, 262–7 (2009). 19676137 10.1515/cclm.2009.061

[b16] Park H. S. *et al.* Dipeptidyl peptidase 10, a novel prognostic marker in colorectal cancer. Yonsei Med J 54, 1362–9 (2013). 24142639 10.3349/ymj.2013.54.6.1362 PMC3809881

[b17] Jerng H. H., Kunjilwar K. & Pfaffinger P. J. Multiprotein assembly of Kv4.2, KChIP3 and DPP10 produces ternary channel complexes with ISA-like properties. J Physiol 568, 767–88 (2005). 16123112 10.1113/jphysiol.2005.087858 PMC1464192

[b18] Jerng H. H., Lauver A. D. & Pfaffinger P. J. DPP10 splice variants are localized in distinct neuronal populations and act to differentially regulate the inactivation properties of Kv4-based ion channels. Mol Cell Neurosci 35, 604–24 (2007). 17475505 10.1016/j.mcn.2007.03.008 PMC3674967

[b19] Cotella D. *et al.* N-glycosylation of the mammalian dipeptidyl aminopeptidase-like protein 10 (DPP10) regulates trafficking and interaction with Kv4 channels. Int J Biochem Cell Biol 44, 876–85 (2012). 22387313 10.1016/j.biocel.2012.02.011

[b20] Djurovic S. *et al.* A genome-wide association study of bipolar disorder in Norwegian individuals, followed by replication in Icelandic sample. J Affect Disord 126, 312–6. 10.1016/j.jad.2010.04.007 20451256

[b21] Singh B. *et al.* A Kv4.2 truncation mutation in a patient with temporal lobe epilepsy. Neurobiol Dis 24, 245–53 (2006). 16934482 10.1016/j.nbd.2006.07.001

[b22] Chen T., Shen X. F., Chegini F., Gai W. P. & Abbott C. A. Molecular characterisation of a novel dipeptidyl peptidase like protein: its pathological link to Alzheimers disease. in *Clin Chem Lab Med*. Vol. 46, A13 (2008).

[b23] van Es M. A. *et al.* Genetic variation in DPP6 is associated with susceptibility to amyotrophic lateral sclerosis. Nat Genet 40, 29–31 (2008). 18084291 10.1038/ng.2007.52

[b24] Allen M. *et al.* Positional cloning of a novel gene influencing asthma from chromosome 2q14. Nat Genet 35, 258–63 (2003). 14566338 10.1038/ng1256

[b25] Girirajan S. *et al.* Refinement and discovery of new hotspots of copy-number variation associated with autism spectrum disorder. Am J Hum Genet 92, 221–37 (2013). 23375656 10.1016/j.ajhg.2012.12.016 PMC3567267

[b26] Strausberg R. L. *et al.* Generation and initial analysis of more than 15,000 full-length human and mouse cDNA sequences. Proc Natl Acad Sci U S A 99, 16899–903 (2002). 12477932 10.1073/pnas.242603899 PMC139241

[b27] Strop P., Bankovich A. J., Hansen K. C., Garcia K. C. & Brunger A. T. Structure of a human A-type potassium channel interacting protein DPPX, a member of the dipeptidyl aminopeptidase family. J Mol Biol 343, 1055–65 (2004). 15476821 10.1016/j.jmb.2004.09.003

[b28] Varki A. Essentials of Glycobiology, (Cold Spring Harbor Laboratory Press, New York., 2009). 20301239

[b29] Rasmussen H. B., Branner S., Wiberg F. C. & Wagtmann N. Crystal structure of human dipeptidyl peptidase IV/CD26 in complex with a substrate analog. Nat. Struct. Biol. 10, 19–25 (2003). 12483204 10.1038/nsb882

[b30] Bjelke J. R. *et al.* Tyrosine 547 constitutes an essential part of the catalytic mechanism of dipeptidyl peptidase IV. J Biol Chem 279, 34691–7 (2004). 15175333 10.1074/jbc.M405400200

[b31] Petrek M. *et al.* CAVER: a new tool to explore routes from protein clefts, pockets and cavities. BMC Bioinformatics 7, 316 (2006). 16792811 10.1186/1471-2105-7-316 PMC1539030

[b32] Krissinel E. & Henrick K. Inference of macromolecular assemblies from crystalline state. J. Mol. Biol. 372, 774–797 (2007). 17681537 10.1016/j.jmb.2007.05.022

[b33] Chien C. H. *et al.* One site mutation disrupts dimer formation in human DPP-IV proteins. Journal of Biological Chemistry 279, 52338–52345 (2004). 15448155 10.1074/jbc.M406185200

[b34] Tang H. K. *et al.* Role of a propeller loop in the quaternary structure and enzymatic activity of prolyl dipeptidases DPP-IV and DPP9. Febs Letters 585, 3409–3414 (2011). 22001206 10.1016/j.febslet.2011.10.009

[b35] Ahn J. H. *et al.* Synthesis and biological evaluation of homopiperazine derivatives with beta-aminoacyl group as dipeptidyl peptidase IV inhibitors. Bioorg Med Chem Lett 18, 6525–9 (2008). 18996694 10.1016/j.bmcl.2008.10.076

[b36] Higueruelo A. P., Jubb H. & Blundell T. L. Protein-protein interactions as druggable targets: recent technological advances. Curr Opin Pharmacol 13, 791–6 (2013). 23735579 10.1016/j.coph.2013.05.009

[b37] Jerng H. H., Dougherty K., Covarrubias M. & Pfaffinger P. J. A novel N-terminal motif of dipeptidyl peptidase-like proteins produces rapid inactivation of KV4.2 channels by a pore-blocking mechanism. Channels (Austin) 3, 448–61 (2009). 19901547 10.4161/chan.3.6.10216 PMC3607289

[b38] Cotella D. *et al.* Impaired glycosylation blocks DPP10 cell surface expression and alters the electrophysiology of Ito channel complex. Pflugers Arch 460, 87–97 (2010). 20354865 10.1007/s00424-010-0824-2

[b39] Bezerra G. A., Dobrovetsky E., Seitova A., Dhe-Paganon S. & Gruber K. Crystallization and preliminary X-ray diffraction analysis of human dipeptidyl peptidase 10 (DPPY), a component of voltage-gated potassium channels. Acta Crystallogr Sect F Struct Biol Cryst Commun 68, 214–7 (2012). 10.1107/S1744309111055230 PMC3274407 22298003

[b40] Leslie A. G. The integration of macromolecular diffraction data. Acta Crystallogr. D62, 48–57 (2006). 10.1107/S0907444905039107 16369093

[b41] Evans P. Scaling and assessment of data quality. Acta Crystallogr. D62, 72–82 (2006). 10.1107/S0907444905036693 16369096

[b42] Long F., Vagin A. A., Young P. & Murshudov G. N. BALBES: a molecular-replacement pipeline. Acta Crystallogr. D64, 125–32 (2008). 10.1107/S0907444907050172 PMC2394813 18094476

[b43] Collaborative Computational Project, N. The CCP4 suite: programs for protein crystallography. Acta Crystallogr. D50, 760–3 (1994). 10.1107/S0907444994003112 15299374

[b44] Emsley P. & Cowtan K. Coot: model-building tools for molecular graphics. Acta Crystallogr. D60, 2126–32 (2004). 10.1107/S0907444904019158 15572765

[b45] Adams P. D. *et al.* The Phenix software for automated determination of macromolecular structures. Methods 55, 94–106 (2011). 21821126 10.1016/j.ymeth.2011.07.005 PMC3193589

[b46] Davis I. W., Murray L. W., Richardson J. S. & Richardson D. C. MOLPROBITY: structure validation and all-atom contact analysis for nucleic acids and their complexes. Nucleic Acids Res 32, W615-9 (2004). 15215462 10.1093/nar/gkh398 PMC441536

