# Supplementary material for: Structure of human dipeptidyl peptidase 10 (DPPY): a modulator of neuronal Kv4 channels
Source: Sci Rep. 2015 Mar 5;5:8769. doi: 10.1038/srep08769 (PMC4350108; doi:10.1038/srep08769)
Supplement: Supplementary Information [file srep08769-s1.pdf]

Structure of human dipeptidyl peptidase 10 (DPPY): a modulator of neuronal Kv4 channels

Gustavo Arruda Bezerra, Elena Dobrovetsky, Alma Seitova, Sofiya Fedosyuk,

Sirano Dhe-Paganon, and Karl Gruber

Supplementary Information

Content: Figures S1-S3

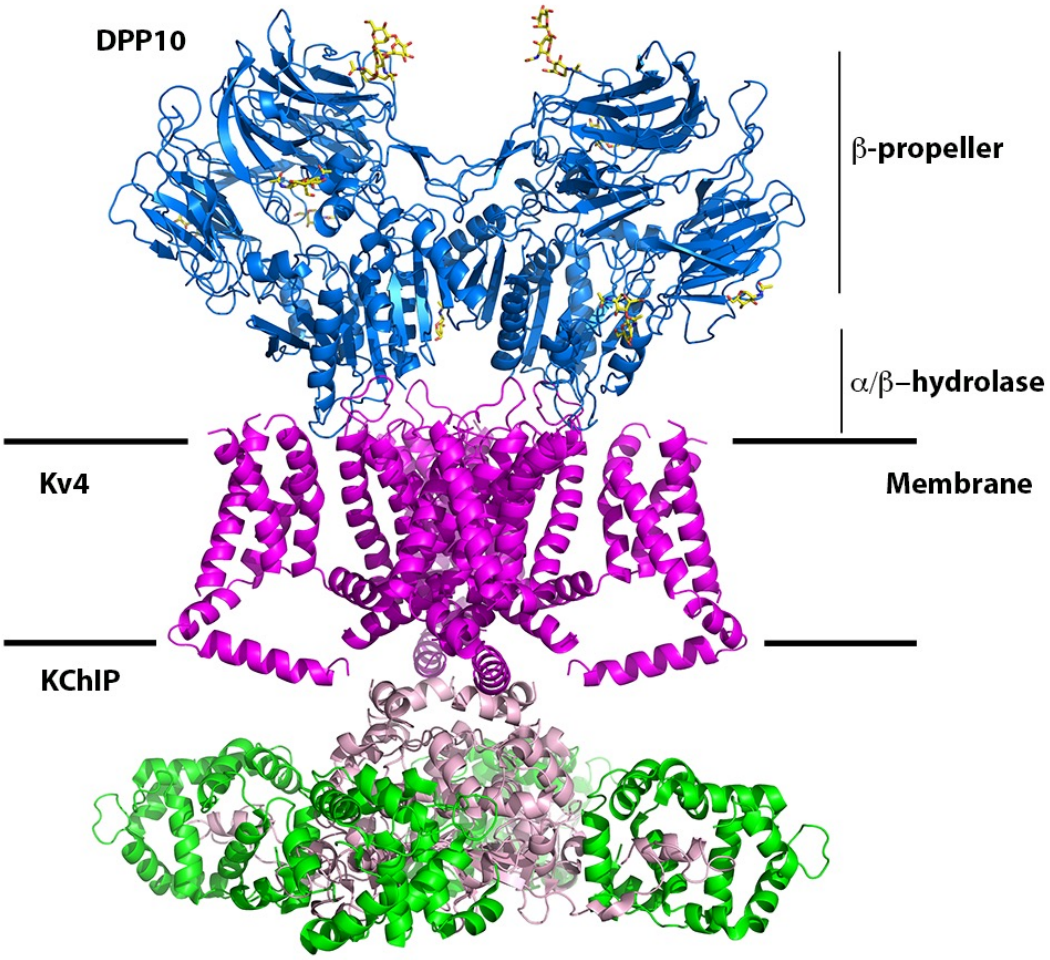

Figure S1: Model of Kv4 channel ternary complex. The model was built by placing DPP10 dimer (shown in blue, carbohydrates as yellow sticks) on the extracellular side of the Kv4 pore subunit model (based on PDB code: 2A79 [1], shown in magenta). Four KChIPs proteins (shown in green) in complex with Kv4.3 cytoplasmic portion (shown in light pink), PDB code: 2NZ0 [2]. For clarity, only 1 dimer of DPP10 is depicted; although 2 dimers are the likely assemble [3]. All proteins are displayed as cartoons.

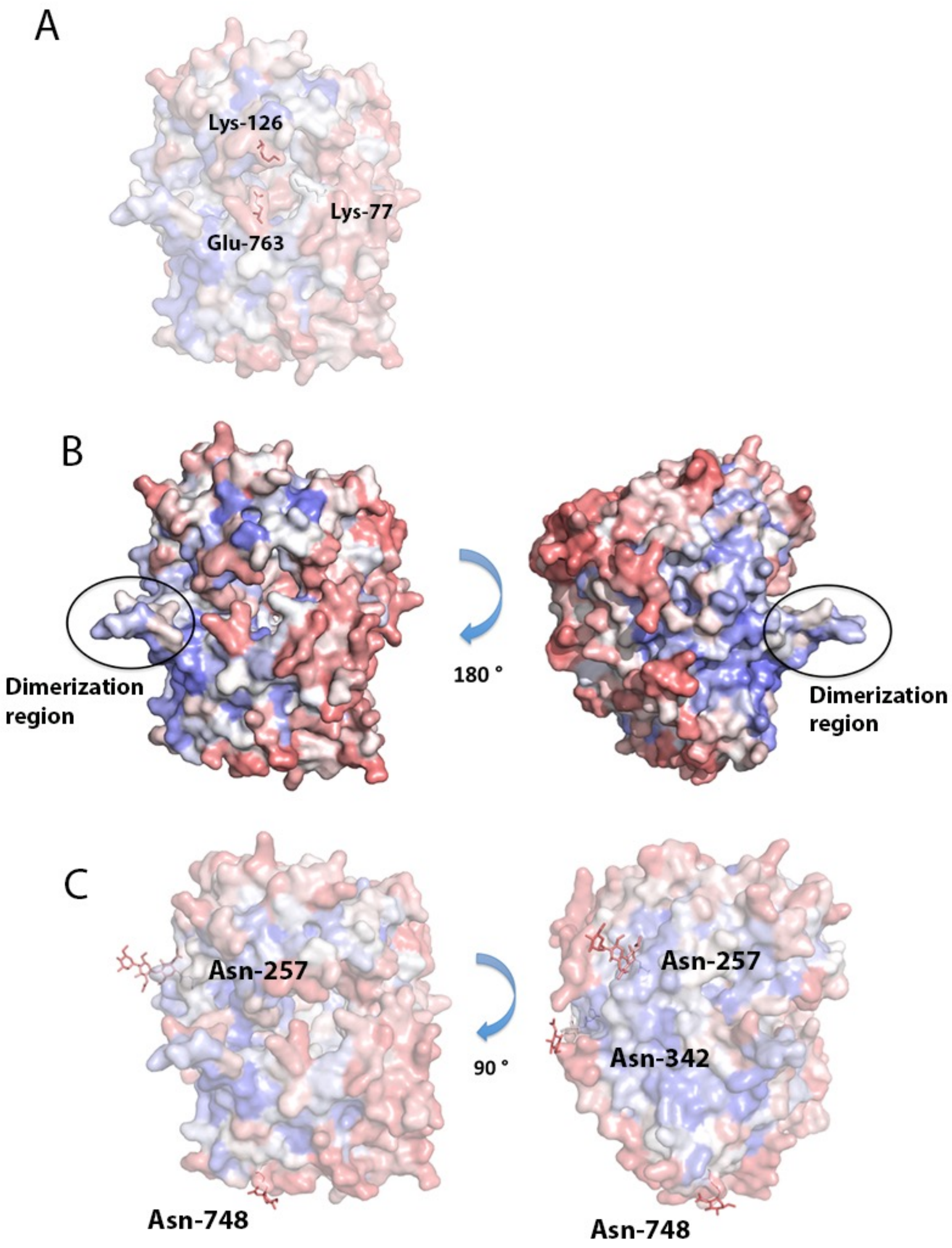

Figure S2: Structure of DPP10 colored according to B-factors. (A) Depiction of residues (shown as sticks) responsible for narrowing the channel entrance to the "active site". **(B)** Illustration of the dimerization region. **(C)** Glycosylation represented as sticks. DPP10 is shown as surface in all items. The color code scale goes from blue (B-factor 31.65 $Å^2$) to red (B-factor 350.10 $Å^2$), the structure average B-factor is 76.33 $Å^2$.

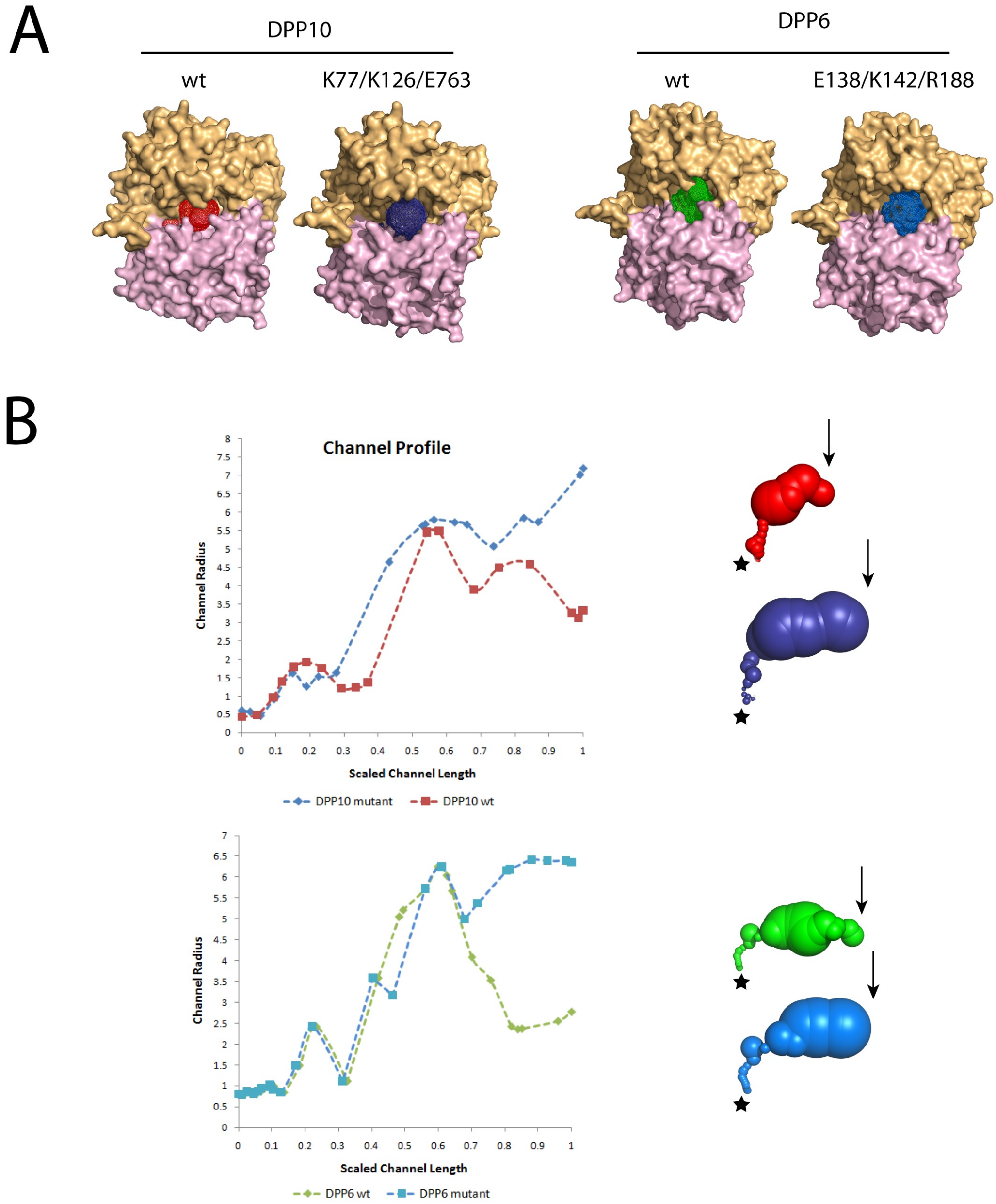

Figure S3: Comparison of the active site access channel in the wild type proteases and *in silico* mutants of DPP10 and DPP6. (**A**) Comparison of surface representation of proteases and their active site access channels for the wild type DPP10 (in red) and DPP6 (in green) with their *in silico* mutants K77/K126/E763 (in dark blue) and E138/K142/R188 (in light blue), respectively. Mutations are done as substitutions of the respective amino acids to alanine. The β-propeller domain is colored in orange and the α/β-hydrolase domain in pink. (**B**) Channel profiles indicating the radius in Å *vs.* the scaled length, starting from the "catalytic triad" position (indicated by a star) towards the protein surface (indicated by an arrow). Surface representation of the channels uses the same coloring scheme as in panel A.

1. Long, S.B., Campbell, E.B. & Mackinnon, R. Crystal structure of a mammalian voltage-dependent Shaker family K+ channel. *Science* **309**, 897-903 (2005).

2. Wang, H. et al. Structural basis for modulation of Kv4 K+ channels by auxiliary KChIP subunits. *Nat Neurosci* **10**, 32-9 (2007).

3. Soh, H. & Goldstein, S.A. I SA channel complexes include four subunits each of DPP6 and Kv4.2. *J Biol Chem* **283**, 15072-7 (2008).
